# Rapid and low-cost detection of six major pediatric enteric pathogens using a closed-tube PCR-invasive reaction assay

**DOI:** 10.3389/fcimb.2026.1841155

**Published:** 2026-06-23

**Authors:** Nan Yang, Jin Zhou, Han Zhang, Yihsuan Huang, Guiping Kong, Yucan Zheng, Zhuo Zhang, Ying Shen, Xiu Han, Zhifeng Liu, Yan Lu

**Affiliations:** 1 Department of Gastroenterology, Children’s Hospital of Nanjing Medical University, Nanjing, China; 2 Department of Clinical Laboratory, Children’s Hospital of Nanjing Medical University, Nanjing, China; 3 School of Basic Medical Sciences, Faculty of Medicine, Yangzhou University, Yangzhou, China

**Keywords:** closed-tube assay, co-infection detection, enteric pathogens, invasive reaction, rapid and low-cost detection

## Abstract

Diarrhea caused by bacterial and viral pathogens remains a major cause of childhood morbidity and mortality, leading to dehydration, malnutrition, and substantial healthcare burdens; rapid and accurate diagnosis is therefore essential for timely treatment. In clinical practice, detection still commonly relies on traditional methods such as stool culture and enzyme-linked immunosorbent assays. These approaches are not only time-consuming and lack sensitivity but also typically require separate, pathogen-specific tests, complicating comprehensive diagnosis. While quantitative PCR (qPCR) offers speed and sensitivity, dye-based methods lack specificity, and fluorescent probe-based assays are costly. To address these limitations, we developed an integrated assay that combines PCR with a sequence-specific invasive reaction (Invader) in a single closed tube. This method detects six major pediatric enteric pathogens: *Shigella* spp., *Vibrio cholerae*, non-typhoidal *Salmonella*, *Campylobacter jejuni*, rotavirus, and enteric adenovirus (types 40/41). The assay demonstrated high sensitivity, with detection limits as low as 2 copies per reaction for each target and exhibited no cross-reactivity. The entire process was completed within 45 minutes. By employing universal Invader probes, we reduced the cost per test to approximately 2 RMB. Validation using both simulated and clinical stool samples suggested the assay’s potential reliability and consistency with standard methods. This closed-tube PCR-Invader assay provides a rapid, cost-effective, and unified platform for the parallel detection of six major diarrheal pathogens in a single test run. It shows promise as a practical alternative to current disparate and pathogen-specific testing strategies, with considerable potential for use in primary healthcare settings and large-scale screening to guide the management of childhood infectious diarrhea.

## 1 Introduction

Infectious diarrhea remains a leading global cause of childhood morbidity and mortality, significantly contributing to malnutrition and impaired growth (Levine et al., 2020). Although mortality has declined, the disease continues to pose a major public health challenge and imposes a substantial economic burden through healthcare costs and lost productivity (GBD 2017 Diarrhoeal Disease Collaborators, 2020). Its etiology is diverse, involving primarily bacteria and viruses, yet clinical symptoms are largely non-specific (Lopez Muñoz et al., 2024). This diagnostic ambiguity frequently leads to empirical antibiotic use, which exacerbates antimicrobial resistance and can prolong illness through inappropriate treatment (Donà et al., 2020; Addis et al., 2023; Blake et al., 2024). Furthermore, mixed infections are common in children, complicating clinical management (Potgieter et al., 2023; Zhang et al., 2025). Consequently, diagnostic strategies capable of rapid, comprehensive, and actionable pathogen identification are essential to guide targeted therapy, improve outcomes, and reduce antibiotic misuse (Jiménez-Jiménez et al., 2025).

Conventional diagnostic methods—including stool culture for bacteria and fungi, microscopic examination for parasites, and enzyme immunoassays for specific pathogens—are often used in parallel due to their distinct target ranges (Sood et al., 2022). However, these approaches are generally time-consuming, lack sensitivity, require specialized expertise, and cannot provide a unified pathogen profile, thereby hindering timely and comprehensive diagnosis (Liu et al., 2020).

Molecular diagnostics, particularly quantitative PCR (qPCR), have enabled faster and more sensitive detection (Li et al., 2024). Among qPCR chemistries, intercalating dye methods such as SYBR Green are cost-effective but prone to false-positive signals from non-specific amplification (Arya et al., 2005). In contrast, probe-based methods like TaqMan offer high specificity by recognizing unique sequences, but the requirement for individually labeled probes for each target substantially increases cost, making simultaneous multi-pathogen testing economically challenging for routine clinical use (Kubista et al., 2006).

The invasive reaction (Invader assay) offers an alternative for sequence-specific detection. It employs a structure-specific flap endonuclease (FEN-1) that recognizes a three-base overlapping structure formed by two target-specific oligonucleotides hybridized to the DNA template. Cleavage releases a universal “flap” sequence, which then triggers a secondary reaction with a common fluorescent hairpin probe (Olivier, 2005). This design allows different targets to be detected using the same universal reporter, requiring only changes in the sequence-specific probe pair, thereby achieving high specificity at a low per-target cost. However, the intrinsic sensitivity of the Invader reaction is limited and necessitates coupling with an upstream amplification step (Sheng et al., 2019). Previous integrations, such as with loop-mediated isothermal amplification (LAMP), often required open-tube steps, raising contamination risks (Lu et al., 2017). Other studies combining qPCR with Invader needed specially modified probes to prevent interference, which added complexity and cost (Shan et al., 2023).

To address these limitations, we developed a novel, cost-effective detection system that seamlessly integrates PCR amplification with Invader-based specific detection in a closed-tube format. The core innovation is a two-stage, high-annealing-temperature PCR protocol that uses temperature to precisely orchestrate sequential amplification and detection phases within the same tube. This eliminates the need for probe modifications and maintains a contamination-free workflow. By applying this standardized reaction format to a panel of six key pediatric diarrheal pathogens—*Shigella* spp., *Vibrio cholerae*, non-typhoidal *Salmonella*, *Campylobacter jejuni*, rotavirus, and enteric adenovirus—we established a unified and streamlined detection platform. This system consolidates the detection of disparate pathogens into six parallel reactions run on a standard qPCR instrument, moving away from the paradigm of separate tests. Our study demonstrates that this platform offers a rapid, low-cost, and practical approach to improving diagnostic efficiency in the management of childhood infectious diarrhea.

## 2 Materials and methods

### 2.1 Instruments and reagents

The following instruments were used in this study: One Drop™ OD-1000 UV-Vis spectrophotometer (Nanjing Wuyi Technology Co., Ltd., Nanjing, China); SLAN-96S real-time PCR system (Shanghai Hongshi Technology Co., Ltd., Shanghai, China). The following reagents were purchased from the indicated suppliers: TB Green™ Premix Ex *Taq*™ (TAKARA, Japan); AMV Reverse Transcriptase (Promega, USA); *Taq* DNA Polymerase (Promega, USA); dNTP mix (SBS Genetech, China); EvaGreen dye (20×, Yeasen, China). The 1× Invader Buffer consisted of 10 mmol/L Tris-HCl, 30 mmol/L NaCl, 7.5 mmol/L MgCl_2_·6H_2_O, 0.05% (v/v) Tween-20, 0.005% (v/v) Nonidet P 40, pH 8.5.

### 2.2 Preparation of standard plasmids

Six common pediatric enteric pathogens were selected as targets: *Shigella* spp., *Vibrio cholerae*, non-typhoidal *Salmonella*, *Campylobacter jejuni*, rotavirus (group A), and enteric adenovirus (types 40/41) (Wang et al., 2021). Conserved genes were selected based on literature review: *ipaH* for *Shigella* (Venkatesan et al., 1989; Bansal et al., 2023), *invA* for *Salmonella* (Heymans et al., 2018), *hipO* for *C. jejuni* (Pham et al., 2015; Alarjani et al., 2021), *ctxA* for *V. cholerae* (Igere, 2024; Sun et al., 2025), vp6 for rotavirus (Kumar et al., 2016), and hexon for adenovirus (La Rosa et al., 2015). To ensure broad coverage in primer design, all available full-length sequences of each target gene were retrieved from the NCBI database. The accession numbers of all sequences used for alignment are listed in **Supplementary Table 1**. Homology alignment was performed using ClustalX2, and highly conserved regions were identified for subsequent primer and probe design. For each pathogen, one representative reference sequence containing the selected conserved region was chosen as the template for plasmid construction; the corresponding accession numbers are provided in **Supplementary Table 2**. The conserved nucleic acid sequences were synthesized and inserted into the pUC57 plasmid (Tsingke Biotechnology Co., Ltd., China) to construct recombinant plasmids. The plasmids were transformed into *E. coli* DH5α competent cells, and single colonies were selected on LB agar plates containing ampicillin. Positive clones were cultured, and plasmids were extracted using a Plasmid Mini Kit (TIANGEN, China). The sequences were verified by Sanger sequencing (Tsingke Biotechnology Co., Ltd., China). Plasmid DNA concentration was measured using the One Drop™ OD-1000 spectrophotometer, and copy numbers were calculated using the formula: copies/µL = [6.02 × 10²³ × concentration (mg/L) × 10^-9^]/(fragment length in bp × 660). Each plasmid was serially diluted to 2 × 10^5^ copies/µL and stored at –20 °C until use.

### 2.3 Design of PCR primers and invader probes

For each conserved gene region, a pair of PCR primers (forward and reverse) were designed using Primer 5 software. The design criteria were as follows: primer length 22–30 bp, GC content 40–60%, Tm 70–74 °C. Secondary structures and dimer formation were evaluated using OligoAnalyzer™ (https://www.idtdna.com/calc/analyzer). The upstream probe (UP), downstream probe (DP), and universal hairpin fluorescence probe (HP) for the Invader reaction were designed based on the amplicon sequences of each pathogen using Universal Invader Design Software. *In silico* specificity was confirmed by BLAST analysis of all primer and probe sequences against the NCBI nucleotide database; no significant matches to non-target enteric pathogens were identified. All primers and probes were synthesized and purified by PAGE (Sangon Biotech Co., Ltd., Shanghai, China). The sequences are listed in **Supplementary Table 3**.

### 2.4 Validation of PCR primer amplification

The amplification feasibility of the designed primers was first verified using a conventional qPCR system with SYBR Green dye. The 20-µL reaction mixture contained: 1× TB Green™ Premix Ex *Taq*™, 0.25 µmol/L each of forward and reverse primers, and 1 µL of template DNA. Thermal cycling was performed as follows: 95 °C for 30 s; 50 cycles of 95 °C for 10 s, 55 °C for 20 s, and 72 °C for 30 s (fluorescence acquisition); 68 °C for 10 min; and a melt curve from 65 °C to 95 °C. Amplicons were analyzed by agarose gel electrophoresis.

To verify the compatibility of the PCR with the downstream Invader reaction in a closed-tube format, a two-step amplification was conducted at an annealing temperature of 70 °C using the final PCR-Invader reaction buffer (1× Invader Buffer). EvaGreen dye was used as the indicator. The 20-µL reaction contained: 1× Invader Buffer, 0.25 µmol/L each of forward and reverse primers, 0.25 µmol/L dNTP mix, 0.25 U *Taq* DNA polymerase, and 1 µL template DNA. The thermal cycling program was: 95 °C for 30 s; 50 cycles of 95 °C for 10 s and 70 °C for 30 s (fluorescence acquisition); 68 °C for 10 min; and a melt curve from 65 °C to 95 °C.

### 2.5 Validation of invader probe specificity

The specificity of the Invader probes was validated using the corresponding PCR amplicons as templates. The 20-µL reaction contained: 1× Invader Buffer, 0.25 µmol/L UP, 1.25 µmol/L DP, 0.25 µmol/L HP, 400 U of FEN1 enzyme (prepared in-house, activity verified by a cleavage assay (Zou et al., 2011)), and 1 µL of PCR product. The reaction was incubated at 85 °C for 1 min, followed by incubation at 63 °C for 20 min with fluorescence signals acquired every 30 s.

### 2.6 Optimization of enzyme concentrations for the PCR-invader assay

To minimize potential interference between PCR amplification and Invader cleavage in the closed-tube format, enzyme concentrations were optimized under the most stringent interference condition, i.e., simultaneous amplification and cleavage at the same annealing temperature. *Shigella* was used as the representative target. The reaction mixture (20 µL) contained: 1× Invader Buffer, 0.25 µmol/L each of forward and reverse primers, 0.25 µmol/L UP, 1.25 µmol/L DP, 0.25 µmol/L dNTP mix, 0.25 µmol/L HP, 1 µL template DNA, and varying concentrations of *Taq* DNA polymerase (2.5, 1, 0.5, or 0.25 U) and FEN1 (400, 200, 80, or 40 U). Thermal cycling was performed as follows: 95 °C for 30 s; 25 cycles of 95 °C for 10 s, 70 °C for 30 s, and 63 °C for 60 s (fluorescence acquisition). The optimal enzyme combination was selected based on the highest fluorescence signal in the positive reaction and the absence of signal in the no-template control (NTC).

### 2.7 Establishment of the closed-tube PCR-invader assay

Based on the optimal enzyme concentrations determined in Section 2.6, the sequential PCR-Invader assay was established to enable PCR amplification followed by Invader-based specific detection in a single closed tube. Each target pathogen is amplified and detected in an individual reaction tube. All six reactions are run simultaneously on the same real−time PCR instrument. The 20-µL reaction mixture contained: 1× Invader Buffer, 0.25 µmol/L each of forward and reverse primers, 0.25 µmol/L UP, 1.25 µmol/L DP, 0.25 µmol/L dNTP mix, 0.25 µmol/L HP, 0.25 U *Taq* DNA polymerase, 400 U FEN1, and 1 µL of template DNA. The thermal cycling program was: 95 °C for 30 s; 35 cycles of 95 °C for 10 s and 70 °C for 30 s (PCR amplification); followed by a temperature decrease to 63 °C and incubation at 63 °C for 20 min with fluorescence signals acquired every 30 s (Invader detection). In each run, a positive control and a no−template control were included. The run was accepted only if the positive control showed a clear positive curve and the NTC remained flat.

### 2.8 Specificity of the PCR-invader assay

The specificity of the PCR-Invader assay was evaluated by testing each target plasmid (2 × 10^5^ copies/µL) against the primer/probe sets designed for the other five pathogens. Each reaction was performed using the optimized reaction system and thermal cycling conditions described in Section 2.7. A no-template control (NTC, water) was included as a negative control, and a single-target plasmid was used as a positive control for each respective set.

### 2.9 Sensitivity of the PCR-invader assay

To determine the analytical sensitivity, each standard plasmid was serially diluted to 2 × 10^5^, 10^4^, 10³, 10², 10¹, and 10^0^ copies/µL. A 1-µL aliquot of each dilution was used as the template in the PCR-Invader assay under the conditions described in Section 2.7. Each dilution was tested in two independent replicates. The limit of detection (LOD) was defined as the lowest template concentration that yielded positive results in both replicates. A positive result was defined by a clear fluorescence curve visually distinguishable from the NTC baseline, which is sufficient for qualitative detection given the typically high pathogen loads in acute diarrhea.

### 2.10 Validation with simulated and clinical stool samples

The established PCR-Invader assay was further validated using both simulated and clinical stool samples. Clinical samples included 55 fecal specimens previously identified as *Salmonella*-positive by bacterial culture (Sample 1-55) and 35 fecal specimens confirmed as human rotavirus A-positive by Sanger sequencing (Sample 56-90). Nucleic acids were extracted using a Viral Nucleic Acid Extraction Kit II (Geneaid, Taiwan) according to the manufacturer’s instructions, followed by reverse transcription using AMV Reverse Transcriptase (Promega, USA). Sample collection was approved by the Ethics Committee of Children’s Hospital of Nanjing Medical University (Ethics approval No. 202110086-1). The Ethics Committee waived the requirement for written informed consent because the study used anonymized residual fecal specimens that had been collected during routine clinical care and were analyzed retrospectively.

Due to the limited availability of clinical samples for the other four pathogens and to simulate co-infections, simulated samples were prepared by spiking target plasmids into nucleic acid extraction supernatants obtained from healthy human fecal samples. Simulated samples included 5 single-positive samples each for *Shigella* (Sample 91-95), *V. cholerae* (Sample 96-100), *C. jejuni* (Sample 101-105), and adenovirus (Sample 106-110); 2 co-infected samples for *Salmonella* + rotavirus (Sample 111, 112); 2 for *Shigella* + adenovirus (Sample 113, 114); 2 for *V. cholerae* + *C. jejuni* (Sample 115, 116); and 2 triple-infected samples for *Salmonella* + rotavirus + adenovirus (Sample 117, 118). A summary of the validation materials for each pathogen is provided in **Supplementary Table 4**. All samples were tested using the PCR-Invader assay under the conditions described in Section 2.7. A reaction was classified as positive if the fluorescence signal showed a clear and sustained increase during the 20−min detection period and the endpoint fluorescence was at least 5−fold higher than the mean endpoint fluorescence of the no−template controls (NTCs). A reaction was classified as negative if the fluorescence signal remained flat throughout the detection period, comparable to the NTCs.

## 3 Results

### 3.1 Principle of the PCR-invader assay

**Figure 1:**
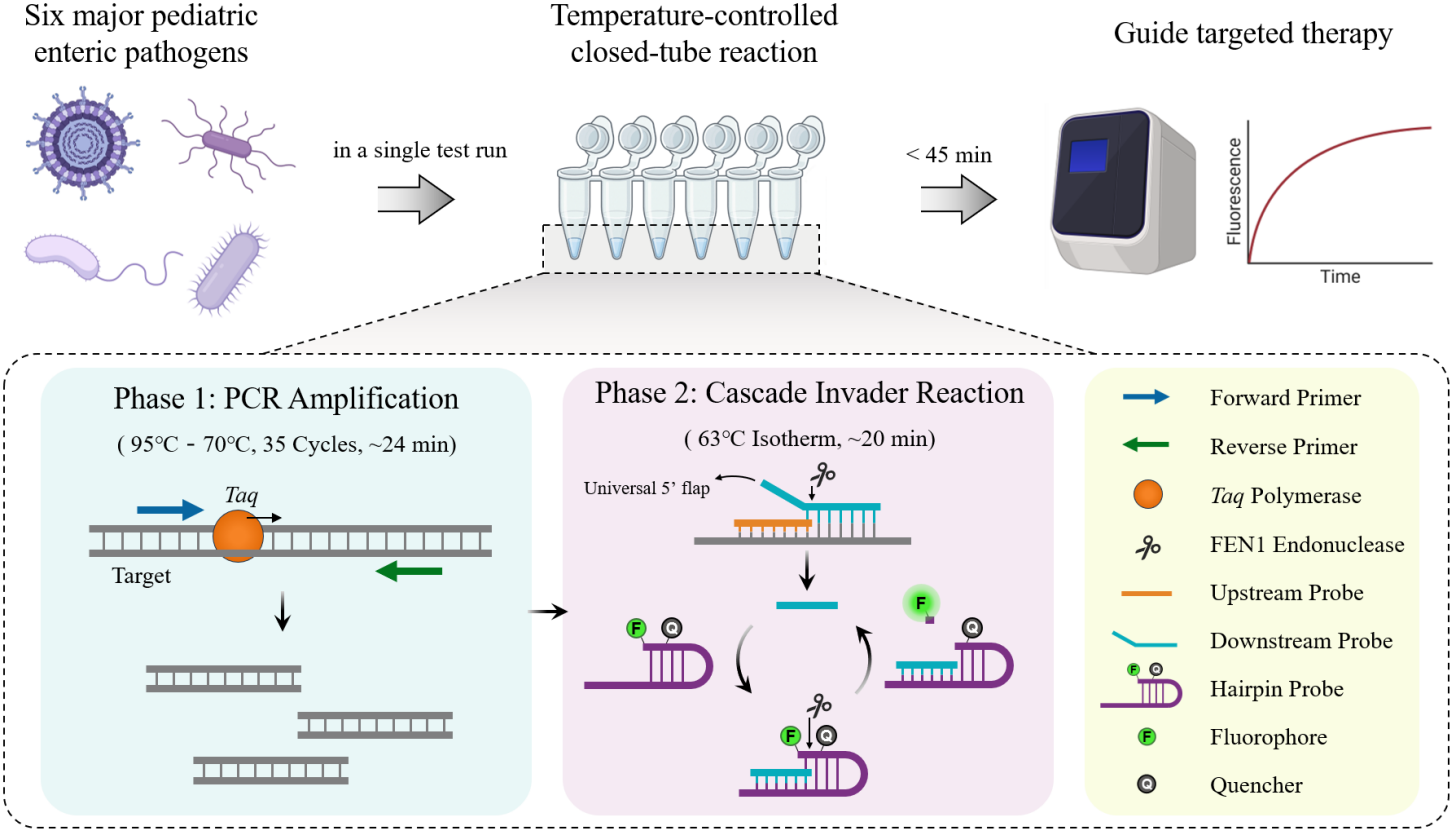
Schematic diagram of the PCR-Invader assay.

The principle of the PCR-Invader assay is illustrated in **Figure 1**. This single closed-tube reaction proceeds sequentially under precise temperature control. The reaction mixture contains both PCR components—forward and reverse primers with *Taq* DNA polymerase—and Invader components, including an upstream probe (UP), a downstream probe (DP), a hairpin probe (HP), and FEN-1 endonuclease. A thermal cycling program first executes 35 cycles of 95 °C and 70 °C to amplify the target DNA specifically, generating abundant amplicons. The temperature is then lowered and held at 63 °C to initiate the Invader reaction. At this temperature, the UP and DP hybridize to the target amplicon. The 3’ terminal nucleotide of the UP overlaps the DP by one base, forming a triplex invasive structure. FEN-1 endonuclease specifically recognizes and cleaves this structure, releasing a universal 5’ flap from the DP. This liberated flap binds to the HP, recreating the invasive structure and triggering a second FEN-1 cleavage event that separates the fluorophore from the quencher to generate a fluorescent signal. This cascade enables rapid signal amplification, with fluorescence quickly reaching a plateau. In the absence of the specific target amplicon, no invasive structure forms, the HP remains intact, and no fluorescence is produced, ensuring high specificity.

### 3.2 Feasibility of PCR primers

**Figure 2:**
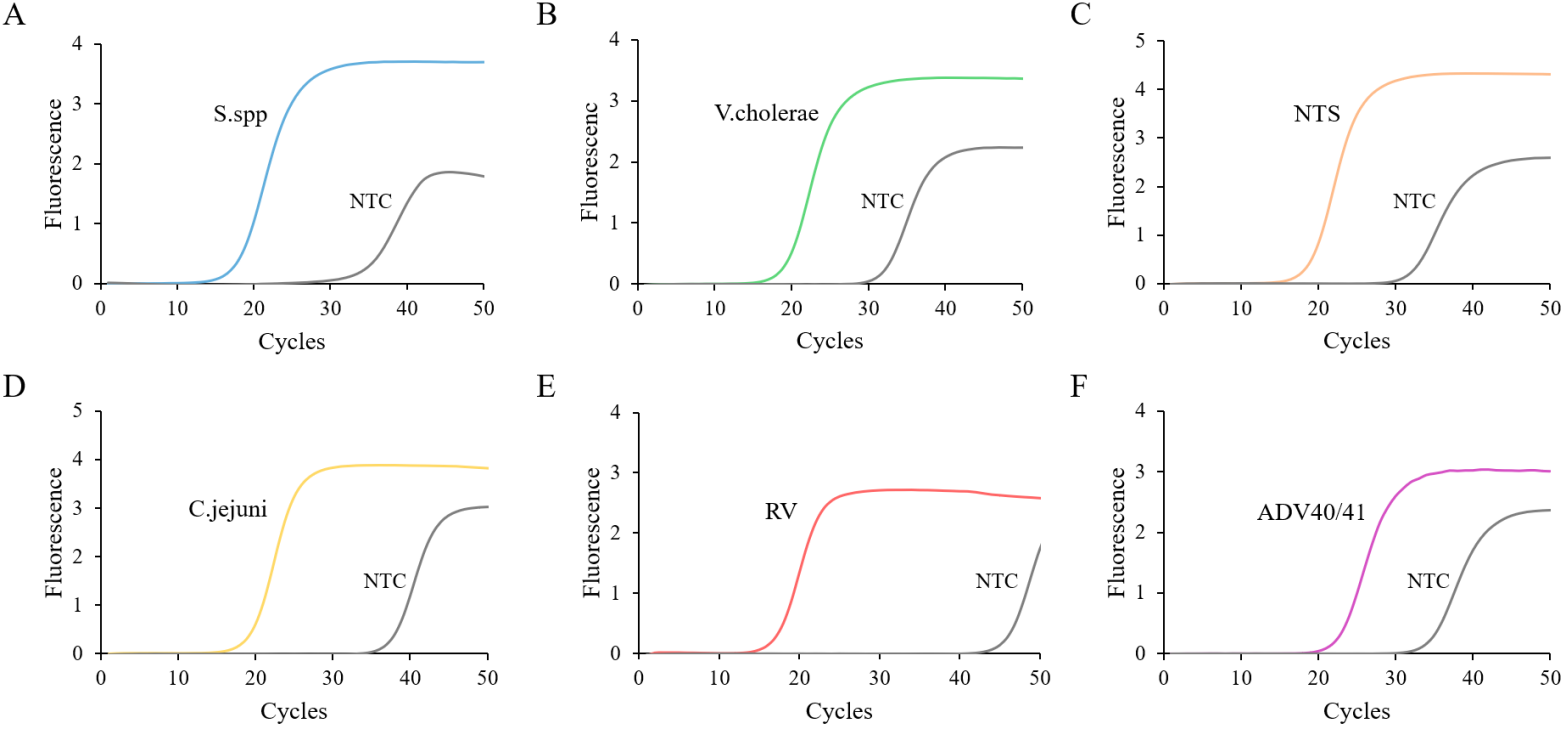
Amplification curves of SYBR Green qPCR for six target DNA fragments. **(A)** *Shigella* spp. **(B)** *Vibrio cholerae*. **(C)** non-typhoidal *Salmonella*. **(D)** *Campylobacter jejuni*. **(E)** Rotavirus (group A). **(F)** Enteric adenovirus (types 40/41). NTC, no-template control.

We first validated the designed primers using a conventional SYBR Green qPCR system to confirm their ability to amplify the target DNA fragments. As shown in **Figure 2**, all six targets exhibited typical amplification curves; positive reactions containing 2×10^5^ template copies initiated amplification around cycle 15, indicating good efficiency. However, all no-template controls (NTCs) also showed amplification beginning around cycle 30. Melt curve analysis (**Supplementary Figure 1**) revealed that the NTC amplification products had distinct Tm values compared to the positive controls, suggesting they were non-specific products such as primer-dimers. Agarose gel electrophoresis (**Supplementary Figure 2**) confirmed this. These results demonstrate that while the primers are functional, SYBR Green-based detection is susceptible to false-positive signals from non-specific amplification, underscoring the need for a more specific detection strategy.

To minimize potential interactions between PCR primers and Invader probes in the single-tube format, we tested a two-step amplification with a high annealing temperature of 70 °C using the PCR-Invader reaction buffer. This temperature exceeds the 63 °C used for the subsequent Invader reaction. The amplification and melt curves are shown in **Supplementary Figures 3**, **4**. Although the positive targets were still amplified, the NTCs exhibited even more pronounced non-specific amplification at this elevated temperature, with complex melt curve profiles. This further highlights the inherent limitation of dye-based methods and reinforces the rationale for employing the highly sequence-specific Invader reaction for downstream detection.

### 3.3 Specificity of the invader probes

**Figure 3:**
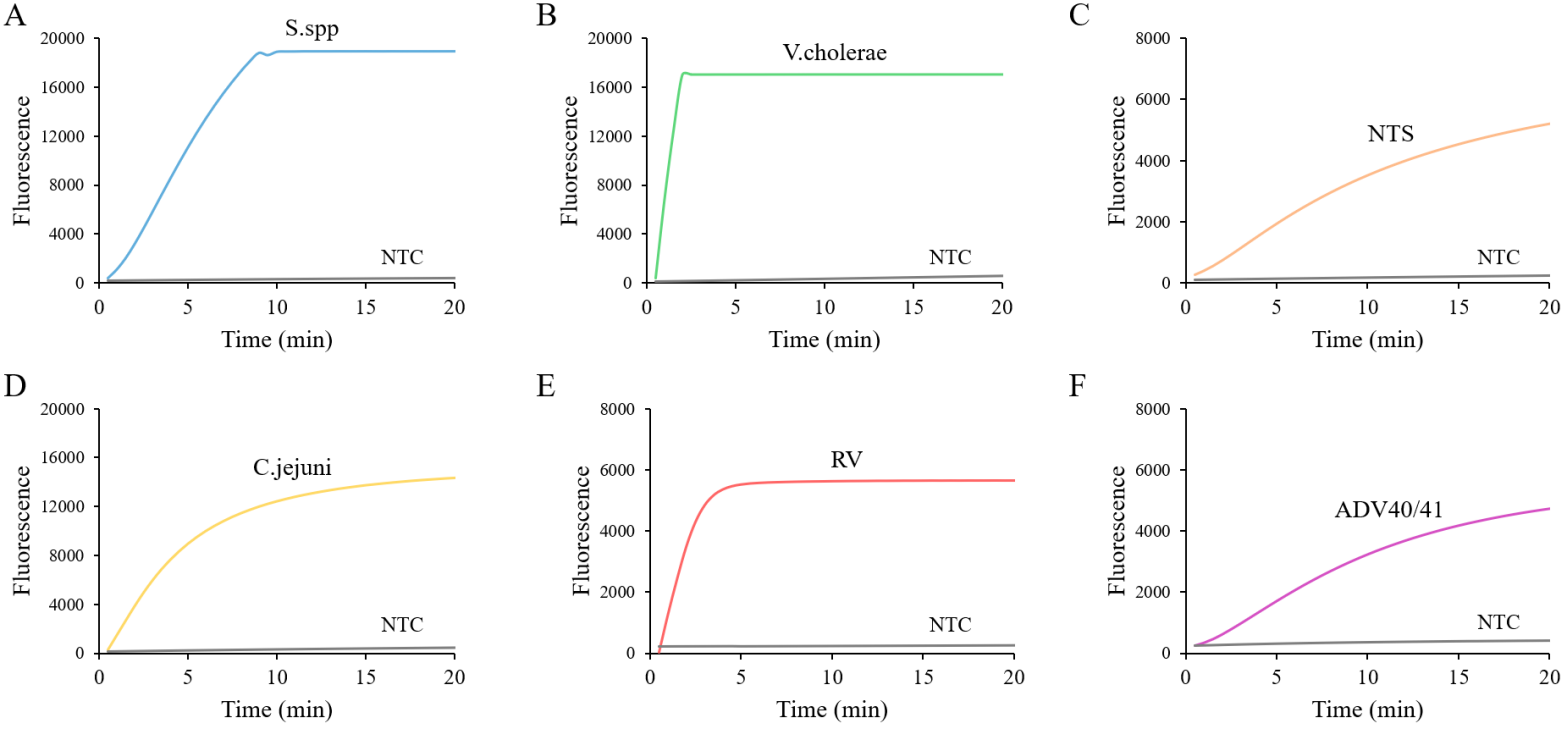
Specificity of the Invader reaction for recognizing target amplicons. **(A)** *Shigella* spp. **(B)** *Vibrio cholerae*. **(C)** non-typhoidal *Salmonella*. **(D)** *Campylobacter jejuni*. **(E)** Rotavirus (group A). **(F)** Enteric adenovirus (types 40/41). NTC, no-template control.

We evaluated the ability of the Invader probes to discriminate between specific and non-specific amplification products. PCR products generated from the 70 °C two-step amplification (section 3.2) served as templates for the Invader reaction. As shown in **Figure 3**, positive PCR products from all six targets—*Shigella*, *V. cholerae*, *C. jejuni*, non-typhoidal *Salmonella*, rotavirus, and adenovirus—successfully triggered the cascade Invader reaction, producing an immediate and linear increase in FAM fluorescence that typically plateaued within 10 minutes (**Figures 3A–F**). In contrast, PCR products from the NTCs generated no detectable fluorescence signal above baseline. These results demonstrate that the Invader probes specifically recognize their cognate target amplicons and ignore non-specific byproducts, confirming the excellent sequence specificity of the reaction.

### 3.4 Optimization of enzyme concentrations for the single-tube PCR-invader assay

**Figure 4:**
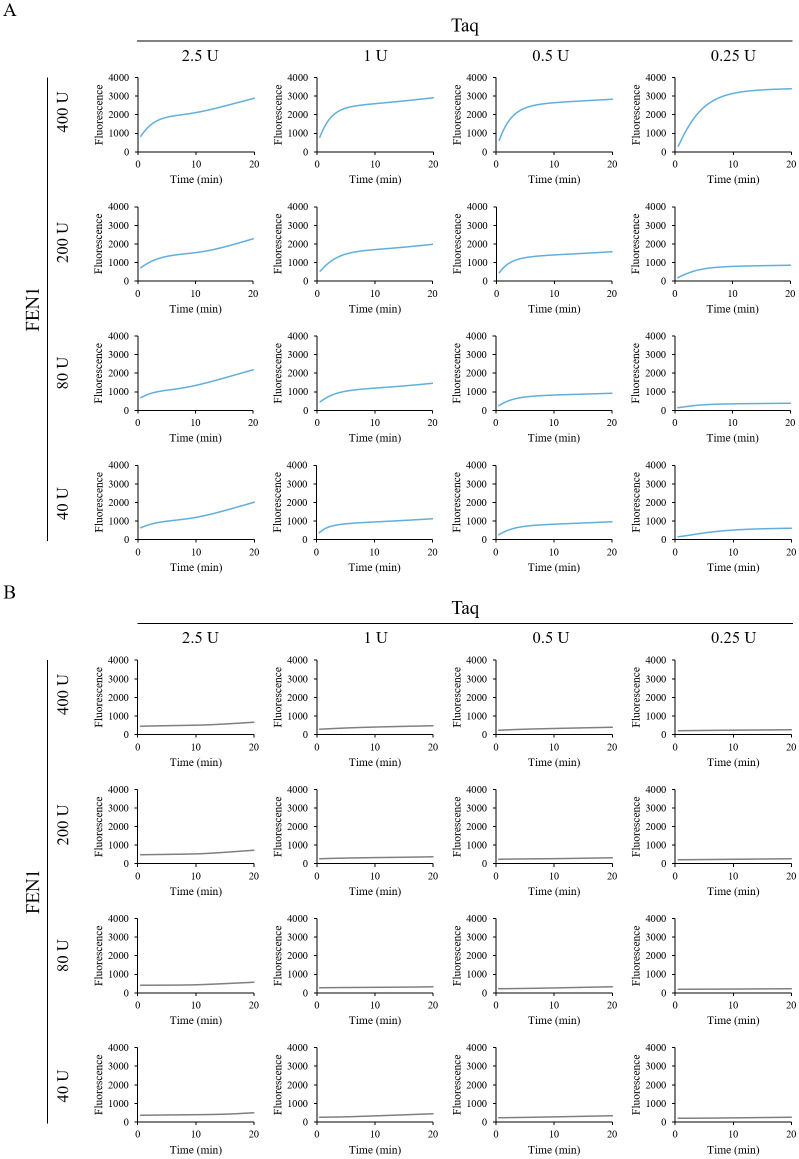
Optimization of *Taq* DNA polymerase and FEN-1 endonuclease concentrations for the closed-tube PCR-Invader assay. Reactions were performed with **(A)** and without **(B)** the *Shigella* target template to assess signal generation and background noise, respectively.

To minimize potential interference between PCR amplification and Invader cleavage in the closed-tube format and to prevent non-specific signal generation, we optimized the concentrations of *Taq* DNA polymerase and FEN-1 endonuclease. Optimization was performed under a stringent condition where both reactions occurred simultaneously at 63 °C, using *Shigella* as a representative target. We tested various combinations of *Taq* polymerase (2.5, 1, 0.5, 0.25 U) and FEN-1 (400, 200, 80, 40 U). As shown in **Figure 4**, both enzyme concentrations significantly influenced reaction kinetics and specificity. The optimal combination was defined as yielding a strong fluorescent signal for the positive control while maintaining a completely flat baseline for the NTC. Based on these criteria, 0.25 U of *Taq* DNA polymerase and 400 U of FEN-1 were selected for all subsequent experiments.

### 3.5 Establishment of the single-tube PCR-invader assay

Using the optimized enzyme concentrations and the sequential thermal protocol, we validated the performance of the single-tube assay for all six pathogens. As shown in **Supplementary Figure 5**, upon entering the 63 °C detection phase, all positive reactions for each pathogen generated a rapid and robust increase in FAM fluorescence that quickly reached a plateau. All NTCs, in contrast, remained completely flat. These results confirm that the sequential PCR-Invader assay functions reliably for all targets, effectively coupling amplification with specific detection in a closed tube and providing excellent signal-to-background performance.

### 3.6 Specificity of the PCR-invader assay

We evaluated the specificity of each detection system by challenging it with high concentrations (2×10^5^ copies/μL) of plasmids from the five non-target pathogens. As shown in **Supplementary Figure 6**, significant fluorescence signals were observed only in the presence of the corresponding target plasmid. No cross-reactivity occurred with any non-target template, demonstrating the high specificity of the PCR-Invader assay for each individual pathogen.

### 3.7 Sensitivity of the PCR-invader assay

**Figure 5:**
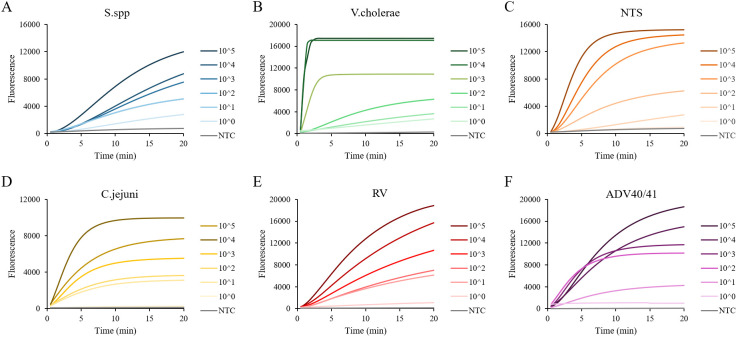
Sensitivity analysis of the PCR-Invader assay for six enteropathogens. **(A)** *Shigella* spp. **(B)** *Vibrio cholerae*. **(C)** non-typhoidal *Salmonella*. **(D)** *Campylobacter jejuni*. **(E)** Rotavirus (group A). **(F)** Enteric adenovirus (types 40/41). NTC, no-template control.

We determined the analytical sensitivity of the PCR-Invader assay using serial dilutions of standard plasmids for each target. As shown in **Figure 5**, the limit of detection (LOD) was 2 copies per reaction for *Shigella* spp. and *V. cholerae*, and 20 copies per reaction for non-typhoidal *Salmonella*, *C. jejuni*, rotavirus, and enteric adenovirus. These results indicate that the developed assay possesses the high sensitivity required to detect low-abundance pathogens in clinical specimens.

### 3.8 Validation with simulated and clinical stool samples

**Figure 6:**
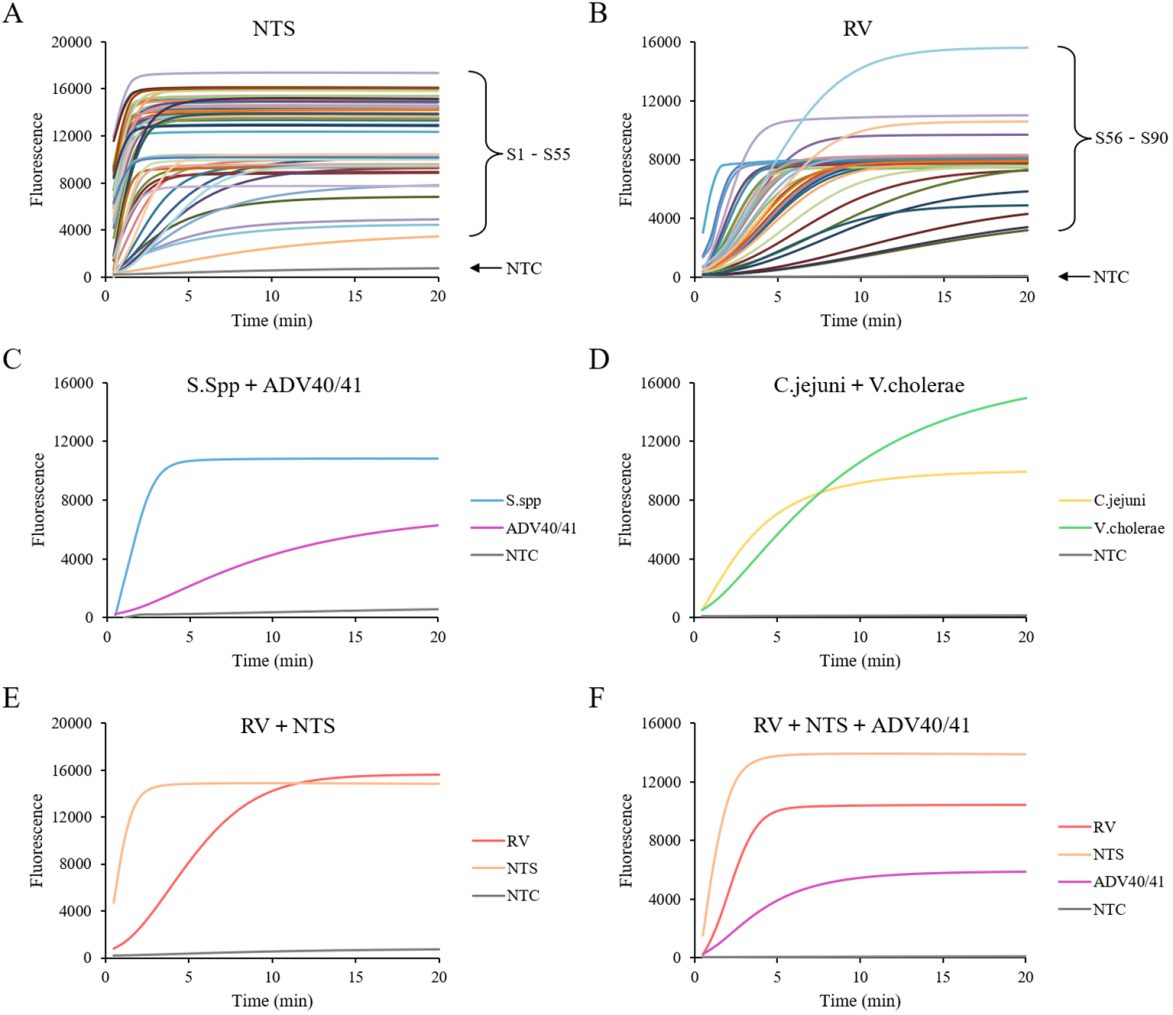
Validation of the PCR-Invader assay using clinical and simulated stool samples. **(A)** 55 clinical samples of non-typhoidal *Salmonella*. **(B)** 35 clinical samples of rotavirus. **(C)** Simulated co-infection sample of *Shigella* spp. and enteric adenovirus (types 40/41). **(D)** Simulated co-infection sample of *Campylobacter jejuni* and *Vibrio cholerae*. **(E)** Simulated co-infection sample of rotavirus and non-typhoidal *Salmonella*. **(F)** Simulated co-infection sample of rotavirus, non-typhoidal *Salmonella*, and enteric adenovirus. S, sample; NTC, no-template control.

We further validated the established PCR-Invader assay using clinical and simulated stool samples. A total of 55 clinical samples previously identified as *Salmonella*-positive by bacterial culture and 35 clinical samples confirmed as rotavirus A-positive by Sanger sequencing were tested. The PCR-Invader assay successfully detected *Salmonella* in all 55 samples (**Figure 6A**) and rotavirus in all 35 samples (**Figure 6B**), showing 100% concordance with the reference methods. The 95% confidence intervals for this concordance are 93.5–100% for *Salmonella* (55/55) and 90.0–100% for rotavirus (35/35) using the Wilson method. For the other four pathogens, testing of simulated positive samples yielded positive results in their respective detection channels, as expected (**Supplementary Figure 7**).

To evaluate the assay’s capability for detecting co-infections, which are common in children with diarrhea, we prepared and tested simulated samples containing two or three pathogens. As shown in the representative results in **Figures 6C–F**, all target pathogens were successfully detected in the mixed-infection samples. These findings confirm that the PCR-Invader assay is accurate for single-pathogen detection and holds potential for diagnosing polymicrobial infections in a single, rapid test.

## 4 Discussion

Infectious diarrhea remains a leading cause of morbidity and mortality in children under five worldwide (GBD 2017 Diarrhoeal Disease Collaborators, 2020; Levine et al., 2020). According to the Global Burden of Disease Study 2016, the six pathogens targeted in our assay – rotavirus, *Shigella* spp., enteric adenovirus, *Vibrio cholerae*, *Campylobacter jejuni*, and non−typhoidal *Salmonella* – collectively account for approximately 84% of diarrheal deaths in children under 5 years worldwide (GBD 2016 Diarrhoeal Disease Collaborators, 2018). In the more recent GBD 2021, these six pathogens remained among the leading causes of diarrheal death in children under 5 years, with population attributable fractions (PAFs) of 35.2%, 24.0%, 23.8%, 7.7%, 7.4%, and 3.5%, respectively (GBD 2021 Diarrhoeal Diseases Collaborators, 2025). Although these PAFs cannot be directly summed owing to frequent co−infections, the six pathogens collectively account for the majority of the attributable diarrheal death burden among young children globally.

Clinical management is complicated by the disease’s diverse etiology, which involves numerous bacterial and viral pathogens that often produce non-specific symptoms (Lopez Muñoz et al., 2024). A rapid, accurate, and comprehensive diagnostic method is therefore essential to guide timely treatment, reduce inappropriate antibiotic use, and improve patient outcomes (Jiménez-Jiménez et al., 2025). Conventional approaches, including stool culture, immunological assays, and microscopy, are often slow, labor-intensive, and lack the sensitivity for comprehensive pathogen detection (Liu et al., 2020; Sood et al., 2022). While qPCR is faster and more sensitive, its two main chemistries have inherent limitations. Dye-based methods like SYBR Green are cost-effective but prone to false positives from non-specific amplification (Arya et al., 2005). Probe-based methods like TaqMan offer high specificity but incur a substantially higher cost per target, making routine multiplex testing economically challenging (Kubista et al., 2006). Although commercial multiplex panels can detect a broad pathogen range, their high cost and need for specialized equipment limit accessibility in resource-limited settings (Osei Sekyere, 2025).

To address these limitations, we developed a novel PCR-Invader assay for the rapid, low-cost, and specific simultaneous detection of six major pediatric enteric pathogens. This system integrates PCR amplification with Invader-based detection in a single closed tube through precise temperature control. As shown in our results, this design streamlines the workflow and overcomes key limitations of previous molecular methods. A major technical challenge in combining PCR with the Invader reaction is preventing interference between the two processes. Conventionally, Invader probes require 3’-modification to block extension by *Taq* polymerase (Xiang et al., 2018; Shan et al., 2023), or the reactions must be physically separated, which increases contamination risk (Ge et al., 2017; Lu et al., 2017). Our assay circumvents this by employing a high-annealing-temperature (70 °C) two-step PCR protocol. At this temperature, the Invader probes cannot stably hybridize, remaining inert during amplification and eliminating the need for chemical modifications. Subsequent cooling to 63 °C halts PCR and initiates the sequence-specific Invader reaction. This temperature-mediated orchestration ensures controlled, sequential steps within a single sealed tube, minimizing contamination and reducing costs.

The integrated design confers practical advantages over existing methods. Compared to traditional stool culture or ELISA, our assay provides results within 45 minutes. It matches or exceeds the speed of many qPCR protocols and commercial panels while benefiting from a closed-tube format (Osei Sekyere, 2025; Serapide et al., 2025). Moreover, the pseudo-first-order kinetics of the Invader reaction suggest potential for even faster detection; monitoring early linear-phase fluorescence could enable reliable interpretation before the reaction plateau, offering a pathway to further reduce total assay time. Crucially, the assay achieves this with a remarkably low cost of approximately 2 RMB per test for six targets. This efficiency stems from using unmodified oligonucleotides and a universal fluorescent hairpin probe, enabled by the temperature-controlled design. The cost per reaction is under 15 RMB for six targets, making it highly competitive for widespread screening, especially in budget-constrained primary care settings. The platform’s reliance on a real-time PCR instrument, a technology now widely accessible globally following diagnostic infrastructure expansion during the COVID-19 pandemic, enhances its implementation potential (Mandhan et al., 2022; Bashir et al., 2024). Existing qPCR instruments in many regions can be readily adapted to run this assay. This synergy with available infrastructure lowers adoption barriers and extends the utility of prior equipment investments, offering a practical choice for laboratories at various levels (Kim et al., 2024).

Analytically, the PCR-Invader assay demonstrates excellent performance. Its limit of detection (2–20 copies/reaction) is comparable or superior to many qPCR assays and is clinically sufficient given the typically high pathogen loads in diarrheal stools (Barletta et al., 2011; Elfving et al., 2014; Wongboot et al., 2018; Xue et al., 2025). The two-tiered specificity from PCR primers and Invader probes ensures robust discrimination, with no cross-reactivity observed in specificity testing. Validation using clinical and simulated samples showed 100% concordance with reference methods, confirming diagnostic accuracy and reliable detection of both single and mixed infections common in pediatric diarrhea.

This study has several limitations. First, the current panel is limited to six pathogens. Although the modular design allows straightforward addition of targets by redesigning primers and probes, the panel must be expanded to include other relevant pathogens to improve diagnostic utility. Second, the number of clinical samples tested for some targets was limited. Specifically, clinical validation was conducted only for *Salmonella* and rotavirus using positive stool specimens. For *Shigella* spp., *V. cholerae*, *C. jejuni*, and adenovirus, only simulated samples were tested. Moreover, no dedicated negative clinical stool samples were tested, so the diagnostic specificity of the assay in actual stool specimens remains unassessed. A larger, multi-center clinical validation is needed to robustly establish the assay’s performance characteristics across diverse populations, and the inclusion of an independent internal amplification control is essential to monitor PCR inhibition and avoid false negatives in future studies. Finally, while mixed-infection detection was demonstrated with simulated samples, further validation with clinical polymicrobial specimens is required to confirm real-world performance in complex diagnostic scenarios. Additionally, our rotavirus assay cannot distinguish between wild-type infection and vaccine strain shedding, as it targets the conserved vp6 gene. Clinicians should therefore interpret positive results together with the child’s age, vaccination history, and symptom severity – a limitation shared by most molecular rotavirus detection methods.

In conclusion, we have established a novel PCR-Invader assay that meets the critical need for a rapid, low-cost, and highly specific method to detect multiple diarrheal pathogens. By integrating amplification and detection through temperature control in a single closed tube, our platform provides a compelling alternative to current diagnostic paradigms. Its simplicity, cost-effectiveness, and robust performance suggest it could be suitable for deployment in resource-limited primary healthcare settings and for large-scale epidemiological screening, with significant potential to improve the management of childhood infectious diarrhea.

## 5 Conclusion

We developed a novel PCR-Invader assay for the rapid, low-cost, and simultaneous detection of six major pediatric enteric pathogens in a closed-tube format. This method employs temperature-controlled sequential reactions, thereby eliminating the need for probe modifications and minimizing contamination risk. The entire procedure is completed within 45 minutes on a standard real-time PCR instrument, a platform that has become widely accessible in clinical laboratories, especially following infrastructure expansion during the COVID-19 pandemic. The assay achieves detection limits of 2–20 copies per reaction at a per-test cost of approximately 2 RMB for all six targets, combining high analytical sensitivity with affordability. Validation using clinical and simulated samples showed 100% concordance with reference methods, suggesting promising diagnostic reliability. This practical, scalable, and equipment-compatible PCR-Invader platform therefore shows strong potential to improve diarrheal disease management in primary healthcare settings and to support large-scale epidemiological surveillance, although further validation using a broader range of clinical specimens is needed. Further validation using clinical samples with mixed infections at different pathogen loads is warranted to fully confirm the assay’s performance in complex diagnostic scenarios.

## Data Availability

The original contributions presented in the study are included in the article/**Supplementary Material**. Further inquiries can be directed to the corresponding authors.
