# Supplementary material for: Rapid and low-cost detection of six major pediatric enteric pathogens using a closed-tube PCR-invasive reaction assay
Source: Front Cell Infect Microbiol. 2026 Jun 23;16:1841155. doi: 10.3389/fcimb.2026.1841155 (PMC13337639; doi:10.3389/fcimb.2026.1841155)
Supplement: Supplementary file 1 [file Supplementaryfile1.pdf]

Supplementary Materials

Rapid and Low-Cost Detection of Six Major Pediatric Enteric Pathogens Using a Closed-Tube PCR-Invasive Reaction Assay

Nan Yang[a,1], Jin Zhou[b,1], Han Zhang[a], Yihsuan Huang[a], Guiping Kong[a], Yucan Zheng[a], Zhuo Zhang[a], Ying Shen[a], Xiu Han[c], Zhifeng Liu[a,*], Yan Lu[a,*]

[a] *Department of Gastroenterology, Children's Hospital of Nanjing Medical University, Nanjing, 210008, China*

[b] *Department of Clinical Laboratory, Children's Hospital of Nanjing Medical University, Nanjing, 210008, China*

[c] *School of Basic Medical Sciences, Faculty of Medicine, Yangzhou University, Yangzhou, 225001, China*

[*] Corresponding authors.

E-mail addresses: zfliu@njmu.edu.cn (Z, Liu), luyan_cpu@163.com (Y. Lu).

[1] These authors have contributed equally to this work.

Table S1. NCBI accession numbers of all full-length sequences retrieved for each target gene for homology alignment.

Shigella		
LC111512.1	LC111492.1	LC111497.1
LC111507.1 S	LC111515.1	LC111517.1
LC111494.1	LC111493.1	LC111505.1
LC111496.1	LC111501.1	LC111508.1
LC111511.1	LC111510.1	LC111509.1
Cholera vibrio		
EU487781.1	AF390572.1	EF158842.1
EF591295.1	FJ748608.1	KU215666.1
MN829555.1	EU546136.1	KY228382.1
KY228383.1	NC_002505.1:c1568114-1567338	NZ_AP014524.1:c1425037-1424261
NZ_AP014524.1:c1418006-1417230	NW_002475684.1:c3621-2845	NC_016445.1:c1042466-1041609
NC_012583.1:c697570-696794	NC_012582.1:c1647342-1646566	NW_001998964.1:c46702-45926
NC_009457.1:c1115923-1115147	NC_009456.1:566372-567148	NC_015209.1:8825-9601
AB699245.1 V	JQ291572.1	AB699245.1
HM042644.1	K02679.1	AF463400.1
AF463401.1		
Salmonella		
LC111482.1	MK017930.1_cds_QBG67159.1_1	MK017931.1_cds_QBG67160.1_1
MK017932.1_cds_QBG67161.1_1	MK017933.1_cds_QBG67162.1_1	MK017934.1_cds_QBG67163.1_1
MK017935.1_cds_QBG67164.1_1	MK017936.1_cds_QBG67165.1_1	MK017937.1_cds_QBG67166.1_1
MK017938.1_cds_QBG67167.1_1	MK017939.1_cds_QBG67168.1_1	MK017939.1_cds_QBG67168.1_1
MK017940.1_cds_QBG67169.1_1	MK017941.1_cds_QBG67170.1_1	DQ644626.1_cds_ABG33961.1_1
MK017942.1_cds_QBG67171.1_1	DQ644628.1_cds_ABG33963.1_1	JF951186.1_cds_AEU60021.1_1
JF951187.1_cds_AEU60022.1_1	JF951188.1_cds_AEU60023.1_1	JF951189.1_cds_AEU60024.1_1
U43237.1_cds_AAC45021.1_1	U43238.1_cds_AAC45022.1_1	U43239.1_cds_AAC45023.1_1
U43240.1_cds_AAC45024.1_1	U43241.1_cds_AAC45025.1_1	U43242.1_cds_AAC45026.1_1
U43243.1_cds_AAC45027.1_1	U43244.1_cds_AAC45028.1_1	U43245.1_cds_AAC45029.1_1
U43244.1_cds_AAC45028.1_1	U43245.1_cds_AAC45029.1_1	U43246.1_cds_AAC45030.1_1
U43247.1_cds_AAC45031.1_1	U43248.1_cds_AAC45032.1_1	U43249.1_cds_AAC45033.1_1
U43250.1_cds_AAC45034.1_1	U43251.1_cds_AAC45035.1_1	U43252.1_cds_AAC45036.1_1
U43271.1_cds_AAC45055.1_1	U43272.1_cds_AAC45056.1_1	U43273.1_cds_AAC45057.1_1
DQ644615.1_cds_ABG33950.1_1	DQ644616.1_cds_ABG33951.1_1	DQ644617.1_cds_ABG33952.1_1
DQ644618.1_cds_ABG33953.1_1	DQ644619.1_cds_ABG33954.1_1	DQ644620.1_cds_ABG33955.1_1
DQ644621.1_cds_ABG33956.1_1	DQ644622.1_cds_ABG33957.1_1	DQ644623.1_cds_ABG33958.1_1
DQ644624.1_cds_ABG33959.1_1	DQ644625.1_cds_ABG33960.1_1	DQ644627.1_cds_ABG33962.1_1
DQ644629.1_cds_ABG33964.1_1	DQ644630.1_cds_ABG33965.1_1	DQ644631.1_cds_ABG33966.1_1

Campylobacillus jejuni					
AY168301.1		NC_002163.1:c919731-918580		Z36940.1	
AY168302.1		KP164636.1		KP164635.1	
KP164633.1		KP164632.1		KP164631.1	
KP164630.1		KP164626.1		KF541295.1	
KF541296.1					
**Rotavirus**					
KU861460.1	KU861449.1	KU861438.1	KU861405.1	KU861394.1	KU861383.1
MT633349.1	MT633347.1	MT633346.1	MT633345.1	MT633343.1	MT633342.1
MT633341.1	MT633340.1	MT633338.1	MT633337.1	MT633336.1	MT633335.1
MT633334.1	MT633333.1	MT633332.1	MT633331.1	MT633330.1	MT633329.1
MT633328.1	MT633327.1	MT633326.1	MT633325.1	MT633324.1	MT633323.1
MT633322.1	MT633321.1	MT633320.1	MT633319.1	MT633318.1	MT633317.1
MT633316.1	MT633315.1	MT633314.1	MT633313.1	MT633312.1	MT633311.1
MT633309.1	MT633308.1	MT633305.1	MT633304.1	MT633303.1	MT633302.1
MT633301.1	MT633300.1	MT633299.1	MT633298.1	MT633297.1	MT633296.1
MT633295.1	MT633294.1	MT633293.1	MT633291.1	MT633290.1	MT633287.1
MT633288.1	MT633286.1	MT633285.1	MT633284.1	MT633283.1	MT633282.1
MT633280.1	MT633278.1	MT633277.1	MT633276.1	MT633275.1	MT633272.1
MT633271.1	MT633270.1	MT633268.1	MT633267.1	MT633266.1	MT633265.1
MT633264.1	MT633263.1	MT633262.1	MT633261.1	MT633260.1	MT633259.1
MT633258.1	MT633257.1	MT633256.1	MT633255.1	MT633254.1	MT633253.1
MT633252.1	MT633250.1	MT633251.1	MT633247.1	MT633246.1	MT633245.1
MT633244.1	MT633243.1	KT694954.1	KT694998.1	KT695009.1	KT695064.1
KT695086.1	KT695108.1	KT695119.1	EU984108.1	AY787645.1	ON563404.1
ON563394.1	ON563384.1	ON563374.1	ON563363.1	ON563342.1	ON563331.1
ON563320.1	ON563309.1	ON563298.1	ON563294.1	ON992641.1	ON992640.1
ON992639.1	ON992638.1	ON992637.1	ON992636.1	ON992635.1	ON992634.1
ON992633.1	ON992632.1	ON992631.1	ON992630.1	ON992629.1	ON992628.1
ON992627.1	ON992626.1	ON992625.1	ON992624.1	ON992623.1	ON992622.1
ON992621.1	ON992620.1	ON992618.1	ON992607.1	ON992606.1	ON992605.1
ON992604.1	ON992603.1	ON992602.1	ON992601.1	ON992600.1	ON992599.1
ON992598.1	ON992597.1	ON992596.1	ON992595.1	ON992594.1	ON992593.1
ON992592.1	ON992591.1	ON992590.1	ON992589.1	ON992588.1	ON992587.1
ON992586.1	ON992585.1	ON992584.1	ON992583.1	ON992582.1	ON992581.1
ON992580.1	ON992579.1	ON992578.1	ON992577.1	ON992576.1	ON992575.1
ON992574.1	ON992573.1	ON992572.1	ON992571.1	ON992570.1	ON992568.1
ON992567.1	ON992566.1	ON992565.1	ON992564.1	ON992563.1	ON992562.1
ON992561.1	ON992560.1	ON992559.1	ON992558.1	ON992557.1	ON992555.1
ON992556.1	ON992554.1	ON992553.1	ON992552.1	ON992551.1	ON992550.1
ON992549.1	ON992548.1	ON992547.1	ON992546.1	ON992545.1	ON992544.1
ON992543.1	ON992542.1	ON992540.1	ON992539.1	ON992538.1	ON992537.1

ON992536.1	ON992535.1	ON992534.1	ON992533.1	ON992532.1	ON992531.1
ON992530.1	ON992529.1	ON992528.1	ON992527.1	MT276805.1	MZ165492.1
MZ165491.1	MZ165490.1	MZ165489.1	MZ165488.1	MZ165487.1	MZ165485.1
MZ165484.1	MZ165483.1	MN106131.1	MN106130.1	MN106129.1	MN106128.1
MN106127.1	MN106126.1	MN106125.1	AB733132.2	KF907300.1	KF907299.1
KF907298.1	KJ721696.1	HQ609561.1	HQ609562.1	LC546081.1	LC546079.1
KU887650.1	KU887649.1	AB022768.1	EU805774.2	KJ820907.1	KJ820895.1
KJ820884.1	KJ820873.1	KJ820862.1	KJ820851.1	KJ820840.1	KJ820829.1
MW331506.1	MZ407480.1	MZ407469.1	MZ407458.1	MZ407447.1	MZ407436.1
MZ407425.1	MZ407392.1	MZ407381.1	MZ407370.1	MZ407337.1	MZ407326.1
MZ407315.1	MZ407304.1	MZ407293.1	MZ407282.1	MZ407271.1	MZ407260.1
MZ407249.1	MW292233.1	MH060122.1	MH060112.1	MH060102.1	MH060082.1
KY937206.1	KY497555.1	KU925787.1	KU248432.1	KU356666.1	KU356655.1
KU356644.1	KU356633.1	KU356622.1	KU356611.1	KU356600.1	KU356589.1
KU356578.1	KU248420.1	KU248409.1	KU248387.1	KU248376.1	KU199285.1
KU199274.1	KU363133.1	KF447843.1	HM066159.1	HM066157.1	HM066158.1
HM066156.1	HM066155.1	HM066154.1	HM066153.1	HM066152.1	HM066151.1
HM066150.1	HM066149.1	HM066148.1	HM066146.1	HM066147.1	HM066145.1
HM066144.1	HM066143.1	HM066142.1	HM066141.1	HM066138.1	HM066139.1
HM066137.1	HM066136.1	HM066135.1	HM066134.1	HM066133.1	HM066132.1
HM066131.1	HM066130.1	HM066129.1	HM066128.1	HM066127.1	HM066126.1
EU679388.1	EU679387.1	EU679385.1	EU679384.1	EU679383.1	MW384411.1
FJ747617.1	MW384378.1	MW384356.1	MW384345.1	MW384334.1	MW384279.1
MW384268.1	MW384257.1	MW384235.1	MW384224.1	MW384202.1	HQ738598.2
LC569546.1	LC569545.1				

Adenovirus

AB330121.1_cds_BAG48817.1_1	40X51782.1_cds_CAA36077.1_1	MW088387.1_cds_QZX52034.1_1
MW088386.1_cds_QZX52033.1_1	MW088385.1_cds_QZX52032.1_1	MW088384.1_cds_QZX52031.1_1
MW088383.1_cds_QZX52030.1_1	MW088382.1_cds_QZX52029.1_1	MW088381.1_cds_QZX52028.1_1
MW088380.1_cds_QZX52027.1_1	MW088379.1_cds_QZX52026.1_1	MW088378.1_cds_QZX52025.1_1
MW088377.1_cds_QZX52024.1_1	MW088376.1_cds_QZX52023.1_1	MW088375.1_cds_QZX52022.1_1
MW088374.1_cds_QZX52021.1_1	MW088373.1_cds_QZX52020.1_1	MW088372.1_cds_QZX52019.1_1
MW088371.1_cds_QZX52018.1_1	MW088370.1_cds_QZX52017.1_1	MW088369.1_cds_QZX52016.1_1
MW088368.1_cds_QZX52015.1_1	MW088367.1_cds_QZX52014.1_1	MW088366.1_cds_QZX52013.1_1
MW088365.1_cds_QZX52012.1_1	MW088364.1_cds_QZX52011.1_1	MW088363.1_cds_QZX52010.1_1
MW088362.1_cds_QZX52009.1_1	MW088361.1_cds_QZX52008.1_1	MW088360.1_cds_QZX52007.1_1
MW088359.1_cds_QZX52006.1_1	MW088358.1_cds_QZX52005.1_1	MW088357.1_cds_QZX52004.1_1
MW088356.1_cds_QZX52003.1_1	MW088355.1_cds_QZX52002.1_1	MW088354.1_cds_QZX52001.1_1
MW088353.1_cds_QZX52000.1_1	MW088352.1_cds_QZX51999.1_1	MW088351.1_cds_QZX51998.1_1

KX581722.1_cds_AOT28354.1_1	L19443.1_cds_AAC13967.1_15	MF962531.1_cds_AVP72669.1_1
MF962530.1_cds_AVP72668.1_1	MF962529.1_cds_AVP72667.1_1	MF962528.1_cds_AVP72666.1_1
MF962527.1_cds_AVP72665.1_1	MF962526.1_cds_AVP72664.1_1	MF962525.1_cds_AVP72663.1_1
MF962524.1_cds_AVP72662.1_1	MF962523.1_cds_AVP72661.1_1	MF962522.1_cds_AVP72660.1_1
MF962521.1_cds_AVP72659.1_1	MF962520.1_cds_AVP72658.1_1	MF962518.1_cds_AVP72656.1_1
MF962517.1_cds_AVP72655.1_1	MF962516.1_cds_AVP72654.1_1	MF962515.1_cds_AVP72653.1_1
MF962514.1_cds_AVP72652.1_1	MF962513.1_cds_AVP72651.1_1	MF962512.1_cds_AVP72650.1_1
MF962511.1_cds_AVP72649.1_1	MF962510.1_cds_AVP72648.1_1	MF962509.1_cds_AVP72647.1_1
MF962508.1_cds_AVP72646.1_1	MF962507.1_cds_AVP72645.1_1	MF962506.1_cds_AVP72644.1_1
MF962505.1_cds_AVP72643.1_1	MF962504.1_cds_AVP72642.1_1	MF962503.1_cds_AVP72641.1_1
MF962502.1_cds_AVP72640.1_1		

Table S2. Accession numbers of representative reference sequences containing the highly conserved regions selected for plasmid construction.

Name	Gene	Sequence ID
Shigella	*Ipah*	LC111497.1
Cholera vibrio	*Ctxa*	EU487781.1
Salmonella	*Inva*	LC111482.1
Campylobacillus jejuni	*Hipo*	AY168301.1
Rotavirus	Vp6	KT695119.1
Adenovirus	Hexon	AB330122

Table S3. Oligonucleotide sequences of PCR primers and Invader probes used in this study.

Pathogen	Oligonucleotide	Sequence (5′→3′)
Shigella	F	GTCCATCAGGCATCAGAAGGCCT
	R	GTCACTCCCGACACGCCATAGAAA
	UP	CCCTGGGCAGGGAAATGTC
	DP	ACGGACGCGGAGTCCGCCTCGAAATTC
Vibrio cholera	F	GGGGCATACAGTCCTCATCCAGATG
	R	CGGAGGGAAACCTGCCAATCCA
	UP	AATTGTTCATCAAGCACCCCAAAATT
	DP	ACGGACGCGGAGGAACTCGATACCATCCATAT
Salmonella	F	CGCCGCCAAACCTAAAACCAGC
	R	CCATCGCGTAACAATACTTCCGGCA
	UP	CTACGTAGACGCTCCGCAAGC
	DP	ACGGACGCGGAGTTGAGCTTTTTCCAGATCT
Campylobacillus jejuni	F	GGTGCGATGATGGCTTCTTCGGAT
	R	GCTCCTATGCTTACAACTGCTGAATTTTGAGG
	UP	TCCTATTTATGCTGCTTCTTTGCTTATTGC
	DP	ACGGACGCGGAGTGGCTTTACAAAGCATAGTA
Rotavirus	F	GTTGGACCAGTATTTCCACCAGGCA
	R	GGAGCCATGTCCTTACTTGCAGAGTC
	UP	CCAGATGGTTAGTCTGGTCCTCAT
	DP	ACGGACGCGGAGCTTAATCAACATGCTTCTGAT
Adenovirus	F	TTCGCCCGTGCCACCGATACCTACTT
	R	TTGTCGCCCACGGCCAGCGTAAA
	UP	CGTCAGTCGCTGCGACCC
	DP	ACGGACGCGGAGTGTCTGTGGTTACATCG
Hairpin Probe	(HP)	FAM-TCT/iBHQ1dT/AGCCGGTTTTCCGGCTAAG ACTCCGCGTCCGT

Notes: F, forward PCR primer; R, reverse PCR primer; UP, upstream probe; DP, downstream probe.

Table S4. Validation material summary for each target pathogen.

Pathogen	Validation material type	Confirmation method / Source	Number tested
Non-typhoidal *Salmonella*	Clinical stool samples	Bacterial culture	55
Rotavirus A	Clinical stool samples	Sanger sequencing	35
Shigella spp.	Simulated samples	Plasmid spiked into healthy stool extract	5
Vibrio cholerae	Simulated samples	Plasmid spiked into healthy stool extract	5
Campylobacter jejuni	Simulated samples	Plasmid spiked into healthy stool extract	5
Adenovirus	Simulated samples	Plasmid spiked into healthy stool extract	5
Co-infections (2-3 pathogens)	Simulated samples	Mixed plasmids spiked into healthy stool extract	8

Notes: All simulated samples were prepared using healthy human stool extracts confirmed negative for the six target pathogens by qPCR.

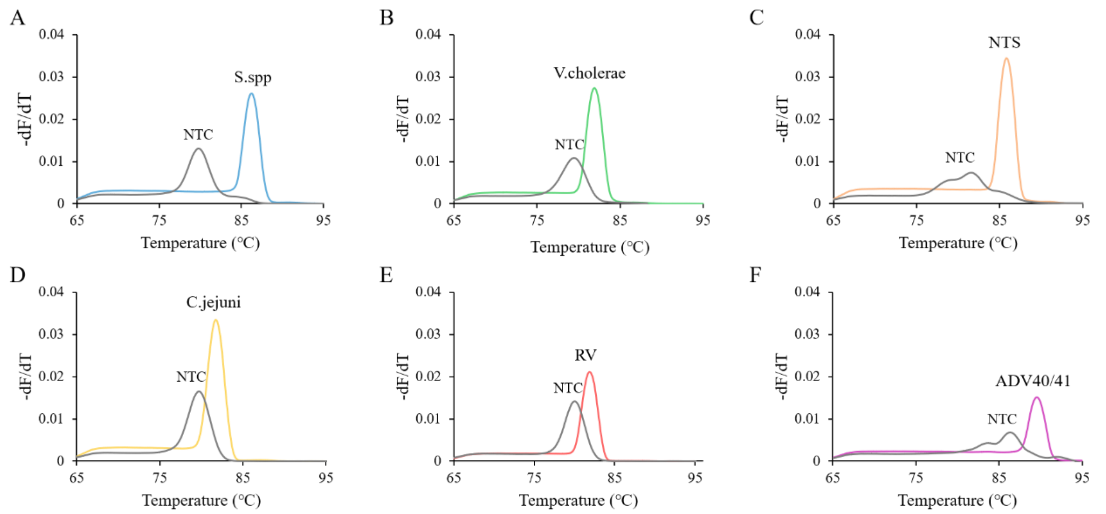

Fig. S1. Melt curve analysis of SYBR Green qPCR products.

A. *Shigella spp.* **B**. *Vibrio cholerae*. **C**. non-typhoidal *Salmonella*. **D**. *Campylobacter jejuni*. **E**. Rotavirus (group A). **F**. Enteric adenovirus (types 40/41). NTC, no-template control.

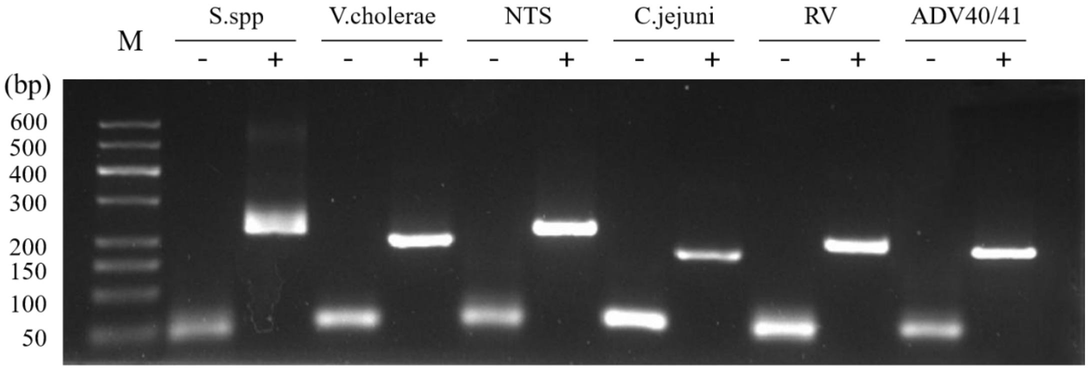

Fig. S2. Agarose gel electrophoresis of SYBR Green qPCR products from six enteropathogens.

Lanes 1-12 show the no-template controls (NTC) and corresponding positive qPCR products for each target in the following order: *Shigella spp.* (NTC, lane 1; positive, lane 2), *Vibrio cholerae* (NTC, lane 3; positive, lane 4), non-typhoidal *Salmonella* (NTC, lane 5; positive, lane 6), *Campylobacter jejuni* (NTC, lane 7; positive, lane 8), rotavirus group A (NTC, lane 9; positive, lane 10), and enteric adenovirus types 40/41 (NTC, lane 11; positive, lane 12). M, DNA marker.

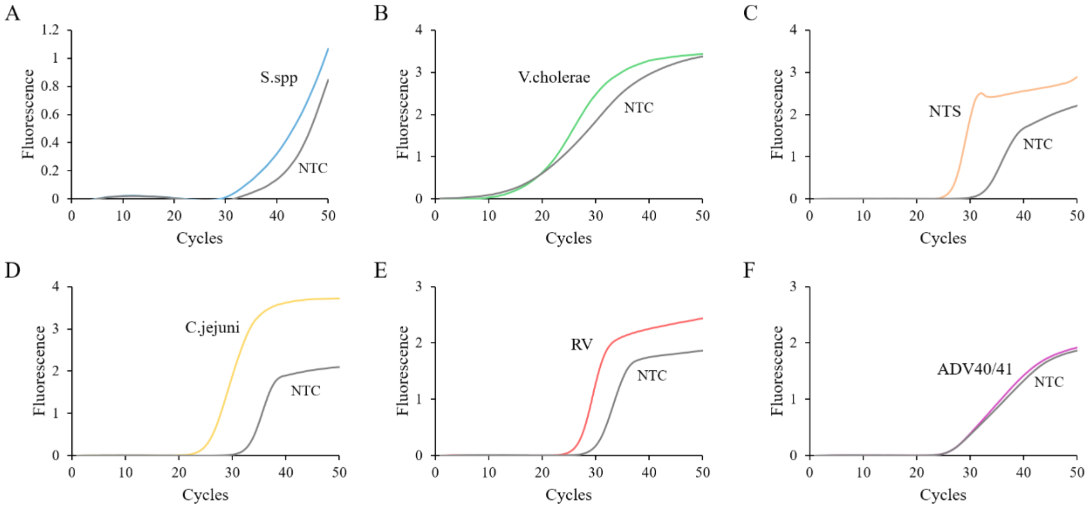

Fig. S3. Amplification curves of the two-step PCR with high annealing temperature (70° C) in PCR-Invader reaction buffer.

A. *Shigella spp*. **B**. *Vibrio cholerae*. **C**. non-typhoidal *Salmonella*. **D**. *Campylobacter jejuni*. **E**. Rotavirus (group A). **F**. Enteric adenovirus (types 40/41). NTC, no-template control.

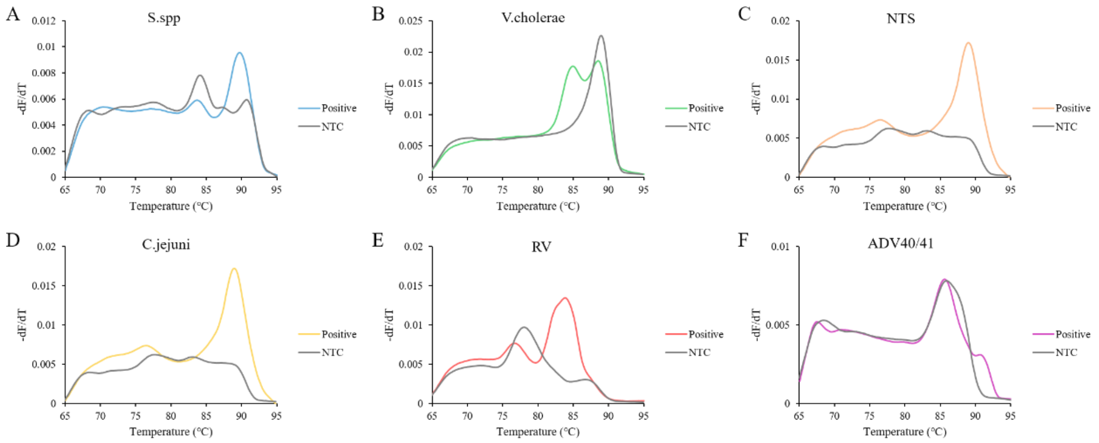

Fig. S4. Melt curve analysis of amplification products from the two-step PCR. **A**. *Shigella spp.* **B**. *Vibrio cholerae*. **C**. non-typhoidal *Salmonella*. **D**. *Campylobacter jejuni*. **E**. Rotavirus (group A). **F**. Enteric adenovirus (types 40/41). NTC, no-template control.

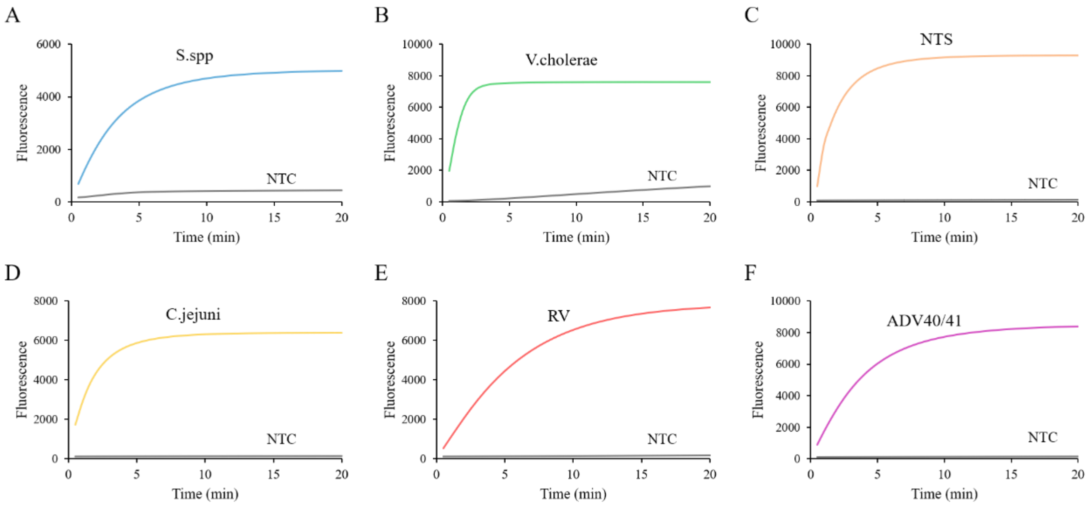

Fig. S5. Validation of the single-tube PCR-Invader assay for six enteropathogens. **A**. *Shigella spp.* **B**. *Vibrio cholerae*. **C**. non-typhoidal *Salmonella*. **D**. *Campylobacter jejuni*. **E**. Rotavirus (group A). **F**. Enteric adenovirus (types 40/41). NTC, no-template control.

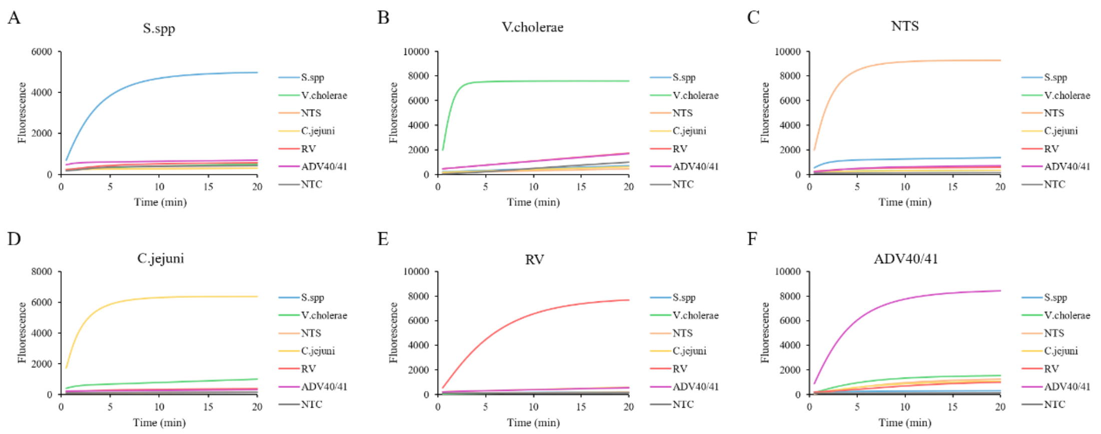

Fig. S6. Specificity evaluation of the PCR-Invader assay for each pathogen. The detection system for each pathogen was tested against high-concentration plasmids (2×10^5 copies/μL) from all six targets: *Shigella spp.* (A), *Vibrio cholerae* (B), non-typhoidal *Salmonella* (C), *Campylobacter jejuni* (D), rotavirus group A (E), and enteric adenovirus types 40/41 (F).

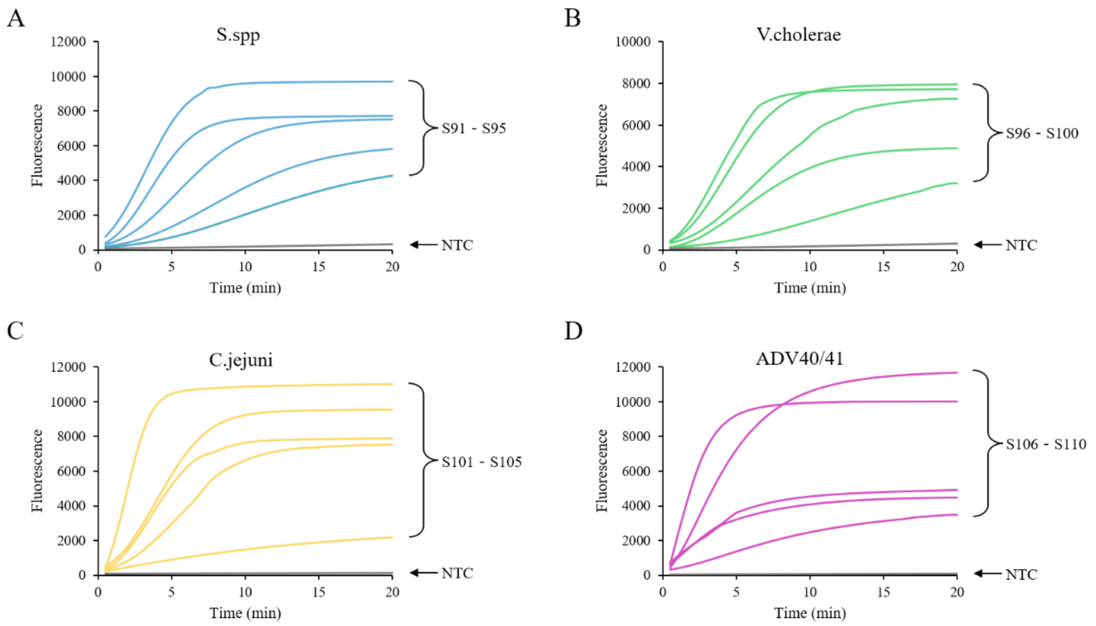

Fig. S7. Detection of simulated positive samples for Shigella, V. cholerae, C. jejuni, and enteric adenovirus.

A. Five simulated samples of *Shigella spp.* **B**. Five simulated samples of *Vibrio cholerae*. **C.** Five simulated samples of *Campylobacter jejuni*. **D**. Five simulated samples of enteric adenovirus types 40/41. NTC, no-template control.
